# Dielectric Model of Carbon Nanofiber Reinforced Concrete

**DOI:** 10.3390/ma13214869

**Published:** 2020-10-30

**Authors:** Zhi-Hang Wang, Jin-Yu Xu, Er-Lei Bai, Liang-Xue Nie

**Affiliations:** 1 Department of Airfield and Building Engineering, Air Force Engineering University, Xi’an 710038, China; wzhsongchen@163.com (J.-Y.X.); songchenwzh@163.com (E.-L.B.); afeuwangzhihang@163.com (L.-X.N.); 2 College of Mechanics and Civil Architecture, Northwest Polytechnic University, Xi’an 710072, China

**Keywords:** carbon nanofiber, concrete, dielectric constant, dielectric model, modification

## Abstract

The formula describing the relationship between the dielectric constant of a composite and the dielectric constants or volume rates of its components is called a dielectric model. The establishment of a cement concrete dielectric model is the basic and key technique for applying electromagnetic wave technology to concrete structure quality testing and internal damage detection. To construct the dielectric model of carbon nanofiber reinforced concrete, the carbon nanofiber reinforced concrete was measured by the transmission and reflection method for dielectric constant ε^,^ and ε^,,^ in the frequency range of 1.7~2.6 GHz as the fiber content was 0, 0.1%, 0.2%, 0.3% and 0.5%. Meanwhile, concrete was considered as a composite material composed of three phases, matrix (mortar), coarse aggregate (limestone gravel) and air, and the dielectric constants and volume rates of each component phase were tested. The Brown model, CRIM (Complex Refractive Index Model) model and Looyenga model commonly used in composite materials were modified based on the experimental data, suitable dielectric models of carbon nanofiber reinforced concrete were constructed, and a reliability check and error analysis of the modified models were carried out. The results showed that the goodness of fit between the calculated curves based on the three modified models and the measured curves was very high, the accuracy and applicability were very strong and the variation rule for the dielectric constant of carbon nanofiber concrete with the frequency of electromagnetic wave could be described accurately. For ε^,^ and ε^,,^, the error between the dielectric constant calculated by the three modified models and the corresponding measured values was very small. For the dielectric constant ε^,^, the average error was maintained below 1.2%, and the minimum error was only 0.35%; for the dielectric constant ε^,,^, the average error was maintained below 3.55%.

## 1. Introduction

The formula describing the relationship between the dielectric constant of a composite and the dielectric constants or volume rates of its components is called a dielectric model [1,2]. Based on the model, the dielectric properties and main influence factors of composites can be understood in detail, so as to support relevant theoretical analysis [3,4,5]. Furthermore, the volume rates of components can be calculated according to the dielectric constant measured by electromagnetic wave nondestructive testing equipment, so as to realize the measurement of quality parameters such as water content and porosity, thus providing technical support for quality inspection and internal damage detection of materials by non-destructive electromagnetic wave technology and broadening the application field of nondestructive electromagnetic wave technology as well [6,7].

Since cement concrete came into being, it has been widely used in industrial, civil construction, national defense military protection and other projects because it is easy to shape, with high long-term strength and low cost [8,9,10,11]. For this, there have been many studies on concrete mix proportion, relevant mechanical properties, durability, constitutive relation and dielectric models [12,13]. Mohamed AB et al. studied the relationship between the dielectric constant of concrete and the moisture content and found that the dielectric constant of concrete increases with the increase in moisture content. The moisture content of a concrete structure can be judged by testing the dielectric constant [14]. Ya P.T. et al. designed and employed a nondestructive method to monitor the early hydration of concrete mixes and found that the dielectric properties of concrete mix can be used as an effective way of studying the hydration progress of concrete during hydration [15]. Hashem A found that dielectric properties are useful parameters for the detection of chloride in concrete material, and the amount of chloride present in concrete can be determined [16]. Thus, the dielectric model of cement concrete is the basis for quality control and subsequent damage detection of concrete structures and has very important theoretical value and engineering significance [17,18,19].

Carbon nanofiber reinforced concrete is a composite of good performance, with ordinary concrete as the base material and carbon nanofiber as the reinforced material [20,21,22,23]. Compared with ordinary concrete, carbon nanofiber reinforced concrete was greatly improved in not only the mechanical properties and durability [22,23,24,25,26] but also the dielectric properties [27,28,29,30,31]. Recently, scholars all over the world have conducted many studies on the dielectric model of cement concrete based on temperature and frequency, with the chloride effect considered, but there are few reports on the dielectric model of carbon nanofiber reinforced concrete [32,33,34,35].

For this, this study utilized the transmission and reflection method to test the dielectric constant of carbon nanofiber reinforced concrete under different fiber content in the frequency range of 1.7 to 2.6 GHz and established a modified dielectric model for the Brown model, CRIM model and Looyenga model based on the experimental data.

## 2. Tests and Results

### 2.1. Materials and Specimen Preparation

The raw materials for the preparation of carbon nanofiber reinforced concrete were as follows: cement (Shaanxi Qinling Cement Group, Xi’an, China), gravel, sand, admixtures (water reducing agent (Shaanxi Haoyu Concrete Admixture Co., Ltd., Xi’an, China), defoamer (Shaanxi Lanxin Chemical Co., Ltd., Xi’an, China)), water and carbon nanofiber (Beijing Dekedaojin Technology Co., Ltd., Beijing, China). The mix proportion of carbon nanofiber reinforced concrete was as shown in Table 1, where PC represents an ordinary concrete specimen without carbon nanofiber as the reference group specimen, while CNFRC1, CNFRC2, CNFRC3 and CNFRC5, respectively, represent nanofiber reinforced concrete specimens with carbon fiber content (v/v) of 0.1%, 0.2%, 0.3% and 0.5%.

**Figure 1:**
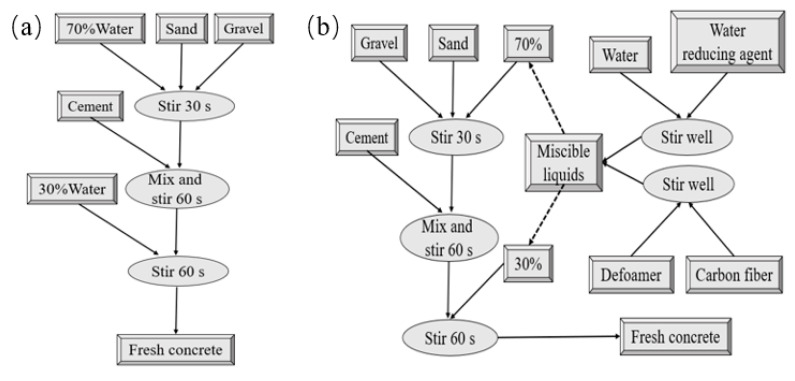
Preparation process of carbon fiber reinforced concrete. (**a**) PC group of specimens; (**b**) CNFCR1, CNFCR2, CNFCR3 and CNFCR5 groups of specimens.

The preparation of carbon nanofiber reinforced concrete was based on the “sand enveloped method “, and the specific process was as shown in Figure 1. The freshly mixed concrete was placed in a steel mold and then on a vibrating table for vibration molding. The specimen was removed after being placed in a room for 24 h, and then we performed 28 days of standard curing. The dimensions of the specimen were 108.22 mm × 53.61 mm × 40.00 mm.

### 2.2. Test Protocol

**Figure 2:**
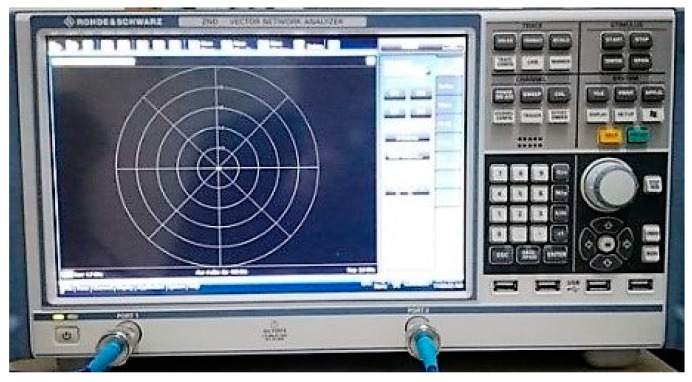
R&S ZND model vector network analyzer.

**Figure 3:**
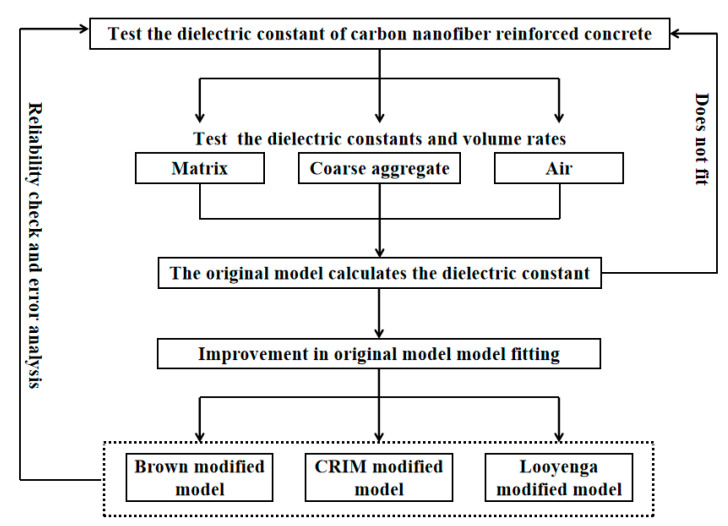
Test protocol flow chart.

The dielectric constant of carbon nanofiber reinforced concrete was tested by the waveguide method (transmission and reflection method). The test system (Shenzhen Wanzhe Instrument Co., Ltd., Shenzhen, China) of the transmission and reflection method was mainly composed of three parts: test fixture, vector network analyzer (as shown in Figure 2) and computer (including data processing software v1.0) The rectangular cavity of the test fixture was filled with the medium to be measured, and the scattering parameter S was obtained by the vector network analyzer after the electromagnetic wave was applied to the medium to be measured. The dielectric constant of the medium to be measured was worked out based on the NRW (Niclson, Ross and Weir) transmission/reflection (T/R) method. The frequency range for the test was 1.7 to 2.6 GHz. Before the test, the equipment should be subjected to a calibration and precision check, but for the same run of tests, the equipment only needed to be calibrated once. The specific test protocol process is shown in Figure 3.

### 2.3. Test Results

**Figure 4:**
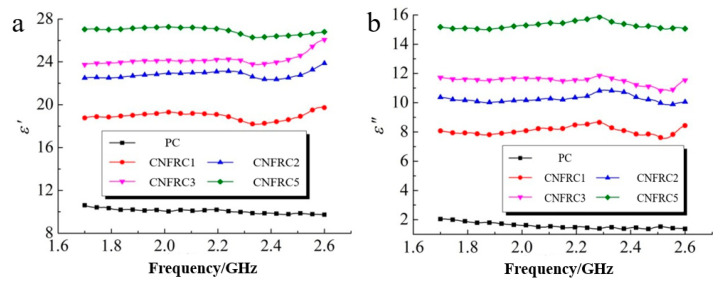
Dielectric constant of carbon nanofiber reinforced concrete (**a**) ε^,^ (**b**) ε^,^.

The test results for the dielectric constant of carbon nanofiber reinforced concrete specimens in the frequency range of 1.7 to 2.6 GHz were as shown in Figure 4. Obviously, for the dielectric constants of carbon nanofiber reinforced concrete, ε^,^ was much higher than ε^,,^. With the increase in carbon nanofiber content, the dielectric constants of carbon nanofiber reinforced concrete, ε^,^ and ε^,,^ increased continuously, because the dielectric constant of carbon nanofiber was much higher than that of the concrete. With the increase in frequency, ε^,^, the dielectric constant of carbon nanofiber reinforced concrete increased slowly at first, then decreased slightly and showed a trend of significant increase at last.

## 3. Dielectric Model

### 3.1. Dielectric Model Introduce

The composite is composed of at least two substances while the main component phases of cement concrete include cement slurry matrix, coarse aggregate, fine aggregate and water. Additionally, inside the cement concrete, there is a small amount of air. The dielectric constant of the composite is not only related to the frequency of external electromagnetic field, but it is also greatly affected by the structure and properties of the material itself, including the dielectric properties, volume rate, etc. In addition, it is also related to the environment of the material, e.g., temperature, humidity, and etc. The influence of these factors on the material may be transformed into a mathematical expression, so as to form a functional relationship between the main factors and the dielectric constant of the material, which is called the dielectric model of composite. At present, there are many dielectric models for composites. In this study, the dielectric model of carbon nanofiber reinforced concrete was constructed based on three basic dielectric models: Brown model (linear model), CRIM model (root-mean-square model) and Looyenga model (cube root model).

In the Lichtenecker–Rother (LR) model, each component phase inside the composite is regarded as a homogeneous medium, and the LR model expression is as follows.

(1) $${\left(\varepsilon_{m}\right)}^{c}=\sum_{i=1}^{n}v_{i}{\left(\varepsilon_{i}\right)}^{c}$$

where *ε_m_* is the dielectric constant of the composite; *ε_i_*, *v_i_*, the dielectric constant and volume of component i for the material; *c*, factor of shape. The value of c depends on specific circumstances. It is related to the properties of the material. In fact, the value of *c* is generally between −1 and 1. Formula (1) can be evolved into the following common dielectric models dependent on different values of *c*:

When *c* = 1, the LR model is evolved into the Brown model, also known as the linear model.

(2) $$\varepsilon_{m}=\sum_{i=1}^{n}v_{i}\varepsilon_{i}$$

When *c* = 0.5, the LR model is evolved into a complex refractive index model (CRIM), also known as the root-mean-square model.

(3) $$\sqrt{\varepsilon_{m}}=\sum_{i=1}^{n}v_{i}\sqrt{\varepsilon_{i}}$$

When *c* = 1/3, the LR model is evolved into the Looyenga model, also known as the cube root model.

(4) $${\left(\varepsilon_{m}\right)}^{1/3}=\sum_{i=1}^{n}v_{i}{\left(\varepsilon_{i}\right)}^{1/3}$$

### 3.2. Construction of Dielectric Model

#### 3.2.1. Concept of Model Construction

For the component phases of carbon nanofiber reinforced concrete, such as cement, sand, gravel, water, fiber, admixture and internal air, when the corresponding dielectric constants are known, the dielectric constant of concrete can be calculated based on the dielectric model of composite. In fact, dielectric constants of materials such as fibers and admixtures are extremely difficult to obtain. At the same time, due to factors such as pouring mode, curing conditions and external environment, it is difficult to determine the hydration degree of cement, and thus, for the formed concrete, the volume rates of some phases (e.g., cement, water and admixtures) are difficult to measure. Therefore, it is necessary to make some assumptions about the formed concrete and to eliminate the errors of the specimens to be tested.

Considering that the Brown model (linear model), CRIM model (root mean square model) and Looyenga model (cube root model) are simply extensive models based on a composite, and a large error may be caused if they are used directly, there should be a modification based on the three models, so as to construct an applicable dielectric model of carbon nanofiber reinforced concrete.

The three models regard component phases of the composite as independent ones which do not interfere with each other. This paper considers that after the carbon nanofiber reinforced concrete is formed, the cement, water and admixture will form an independent matrix (mortar) after the physical and chemical reaction. The matrix can be regarded as a single phase. The volume admixture method is used for the fiber, and the content of fiber is only 0.5%. Therefore, the influence of fiber on the dielectric parameters of concrete is negligible. The error exclusion treatment for the specimens is mainly for the moisture inside the specimens. Because it is difficult to accurately measure the moisture content of the concrete specimens at the end of curing, the specimens are dried to remove the free water inside. Therefore, the influence of water on the dielectric constant of the dehydrated specimens may also be neglected. The air phase inside the formed concrete cannot be ignored. Here, it is assumed that all the pores inside the concrete are filled with air. Therefore, after certain assumptions and error elimination treatment, the concrete is composed of three phases: matrix (mortar), coarse aggregate (limestone gravel) and air. Thus, the theoretical dielectric constant of concrete can be simply calculated by the model after the dielectric constants of these three phases and their volume rates in concrete are worked out.

#### 3.2.2. Determination of Dielectric Constants and Volume Rates of Each Component Phase

Generally, air is relatively stable in nature. Its dielectric constant is about 1, and its static conductivity is 0. Since the dielectric parameters of air could be accurately measured with the transmission and reflection method adopted in this study, relevant data from references were cited.

**Figure 5:**
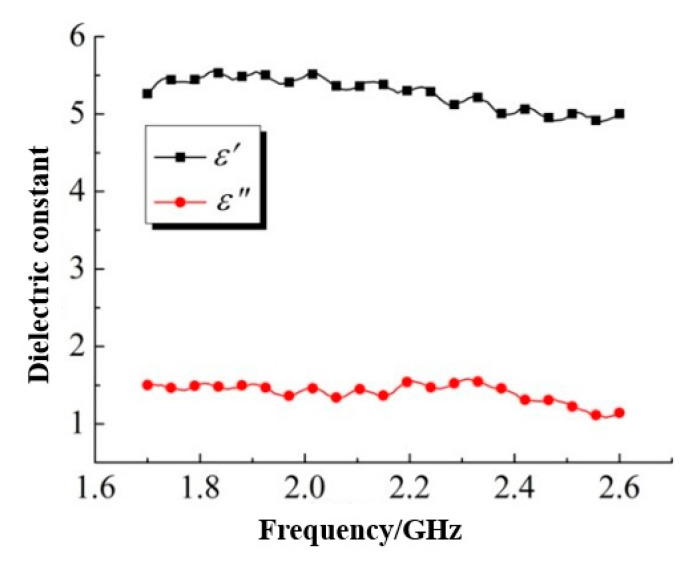
Dielectric constant of limestone gravel.

The coarse aggregate used in this test was limestone gravel. The gravel was cut and polished into specimens with dimensions of 108.22 mm × 53.61 mm × 40.00 mm, which were also tested by the transmission and reflection method, and the test results were as shown in Figure 5. Obviously, for the dielectric constants of limestone gravel, ε^,^ was far higher than ε^,,^. The dielectric constants ε^,^ and ε^,,^ were maintained at about 5.5 and 1.5, respectively, and both of them decreased slightly with the increase in electromagnetic frequency. However, the amplitude of variation was not large on the whole.

**Figure 6:**
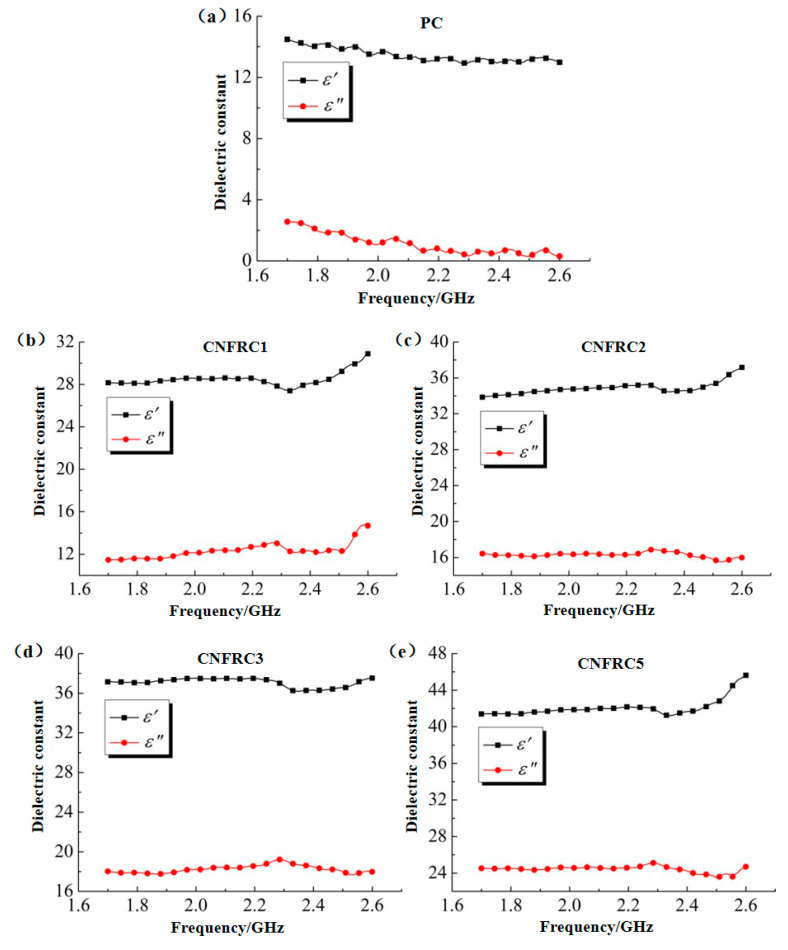
Dielectric constants of cement mortar for groups. (**a**) PC; (**b**) CNFRC1; (**c**) CNFRC2; (**d**) CNFRC3; (**e**) CNFRC5.

In order to obtain a cement mortar with matrix composition the same as the carbon nanofiber reinforced concrete, in this study, a cement mortar of the corresponding components was prepared additionally, and the content of other components in it remained the same, except for coarse aggregate. The test results for dielectric constants of cement mortar for each group were as shown in Figure 6. Obviously, compared with the complex dielectric constant of concrete in the same group as Figure 3, the complex dielectric constants of cement mortar were generally high. The main reason for this was that the complex dielectric constants of limestone gravel were relatively low. In addition, its variation in dielectric constants ε^,^ and ε^,,^ with electromagnetic wave frequency was generally consistent with that of concrete in the same group. With the increase in carbon nanofiber content, the dielectric constants of cement mortar, ε^,^ and ε^,,^, increased continuously, because the dielectric constant of carbon nanofiber was higher than that of the cement mortar. With the increase in frequency, ε^,^ and ε^,,^, the dielectric constants of cement mortar in the PC group, decreased slightly, while in other groups, the dielectric constant ε^,^ of cement mortar first decreased and then increased, and there was no obvious rule for the change in the dielectric constant ε^,,^ of cement mortar.

The volume rate of limestone gravel in each group was calculated as 37.33% according to the mix proportion and the property parameters of limestone gravel. The mercury injection method was used to test the total pore volume for groups of concrete specimens, as shown in Table 2. The void content of specimens in the PC group, CNFC1 group, CNFC2 group, CNFC3 group and CNFC5 group was 6.66%, 5.76%, 5.35%, 4.98% and 5.28%, respectively.

### 3.3. Modification of Dielectric Model

#### 3.3.1. Calculation of Original Model

Dielectric constants of air, coarse aggregate and matrix were recorded as *ε_1_*, *ε_2_* and *ε_3_*, respectively, and the volume rates of the three were recorded as *v_1_*, *v_2_* and *v_3_*.

(1) Brown model

The formula for calculation of the original Brown model is as follows.

(5) $$\varepsilon_{m}=v_{1}\varepsilon_{1}+v_{2}\varepsilon_{2}+v_{3}\varepsilon_{3}$$

**Figure 7:**
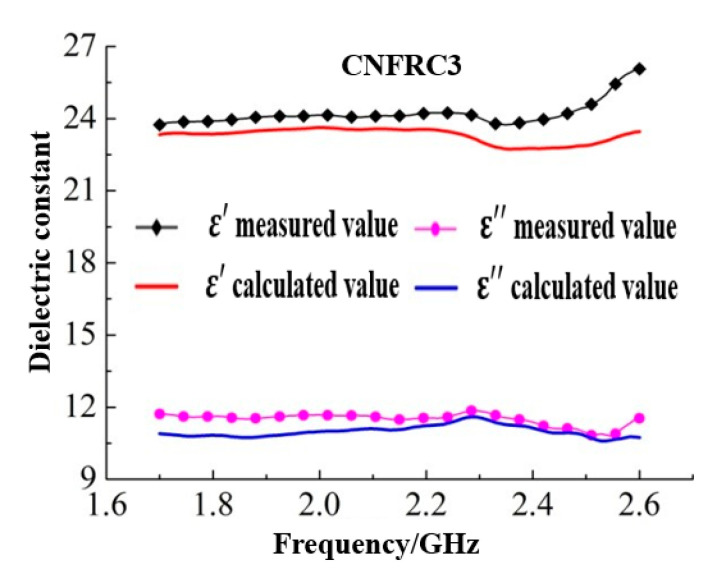
Results of dielectric constants calculated based on Brown model.

The dielectric constants for groups of specimens calculated based on Formula (5) were as shown in Figure 7 (only some of the groups were given).

(2) CRIM model

The formula for calculation of the original CRIM model is as follows.

(6) $$\sqrt{\varepsilon_{m}}=v_{1}\sqrt{\varepsilon_{1}}+v_{2}\sqrt{\varepsilon_{2}}+v_{3}\sqrt{\varepsilon_{3}}$$

**Figure 8:**
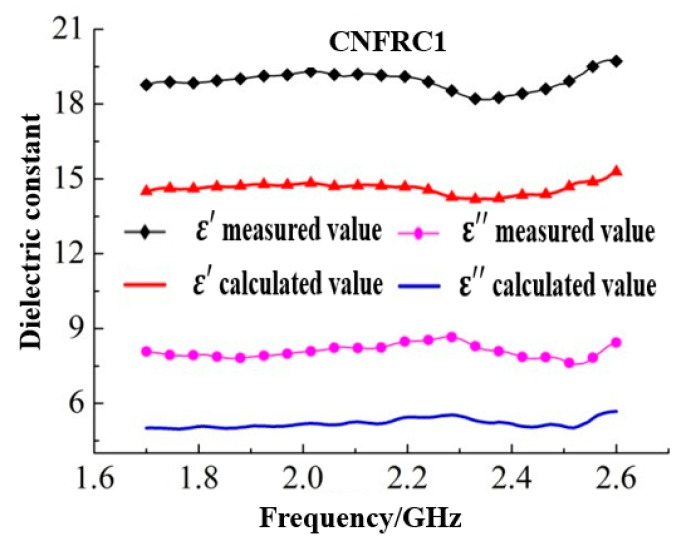
Results of dielectric constants calculated based on CRIM model.

The dielectric constants for groups of specimens calculated based on Formula (6) were as shown in Figure 8 (only some of the groups were given).

(3) Looyenga model

The formula for calculation of the original Looyenga model is as follows.

(7) $${\left(\varepsilon_{m}\right)}^{1/3}=v_{1}{\left(\varepsilon_{1}\right)}^{1/3}+v_{2}{\left(\varepsilon_{2}\right)}^{1/3}+v_{3}{\left(\varepsilon_{3}\right)}^{1/3}$$

**Figure 9:**
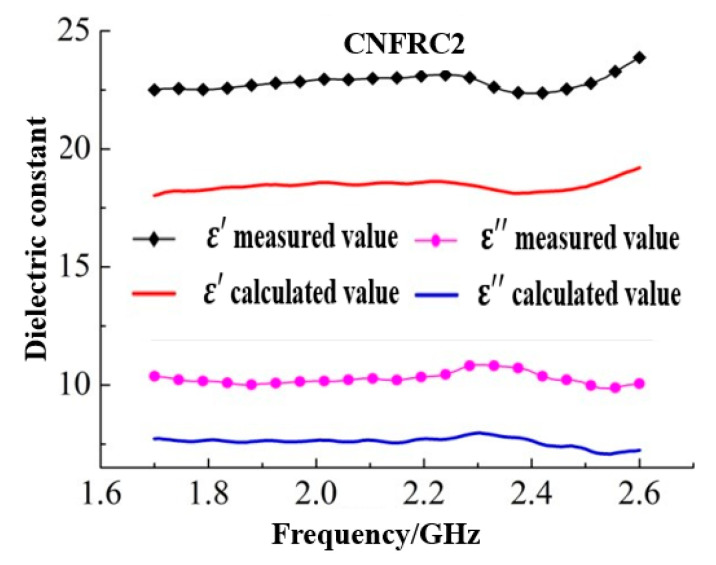
Results of dielectric constants calculated based on Looyenga model.

The dielectric constants for groups of specimens calculated based on Formula (7) were as shown in Figure 9 (only some of the groups were given).

According to Figure 7, Figure 8 and Figure 9, the goodness of fit between the measured results and the calculated results based on the three classical models was low, which indicated that the addition of carbon nanofiber had greatly influenced the dielectric constant of concrete, and the original model did not effectively describe the test results. As shown in Figure 7, Figure 8 and Figure 9, the measured results were generally consistent with the calculated results based on the three classical models in overall change rule. Therefore, to construct a suitable dielectric model of carbon nanofiber reinforced concrete, the three classical models should be optimized and modified respectively.

#### 3.3.2. Improvement in Model Fitting

According to a relevant analysis, the measured values were generally consistent with calculated values based on the three classical models in overall change rule. Therefore, the measured values and calculated values were subjected to linear fitting first, and then the models were modified based on the fitting parameters.

It should be noted that although the rule for dielectric constants of the specimens within the whole test frequency range was obvious, the values fluctuated greatly during the period. Especially for the CNFRC group, the linear fitting between measured values and calculated values were not satisfactory. Therefore, within the whole test frequency range, some representative points reflecting the variation rule for dielectric constants of the specimens were selected for fitting. This may not only improve their fitting degree but also retain the overall rule, thus ensuring the reliability of the data.

**Figure 10:**
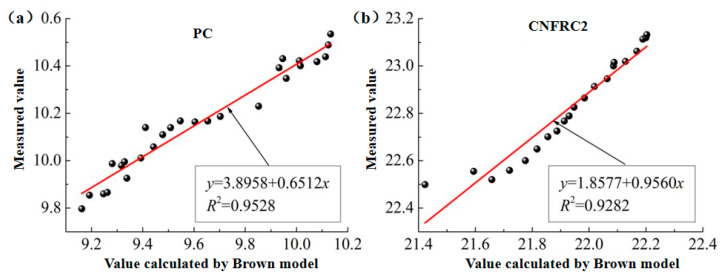
The fitting for relation between measured values and calculated values for ε^,^ based on Brown model. (**a**) PC; (**b**) CNFRC2.

**Figure 11:**
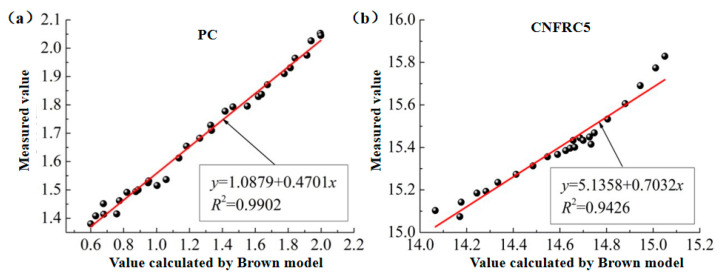
The fitting for relation between measured values and calculated values for ε^,,^ based on Brown model. (**a**) PC; (**b**) CNFRC5.

**Figure 12:**
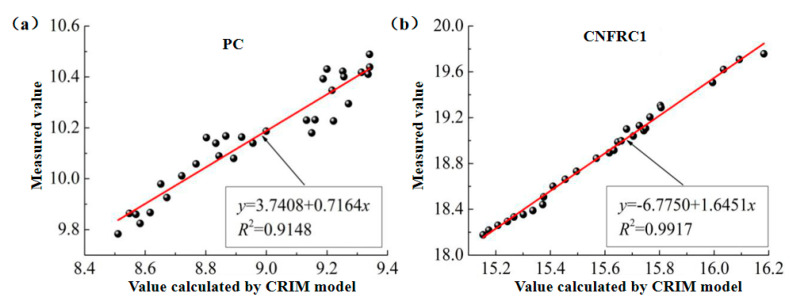
The fitting for relation between measured values and calculated values for ε^,^ based on CRIM model. (**a**) PC; (**b**) CNFRC1.

**Figure 13:**
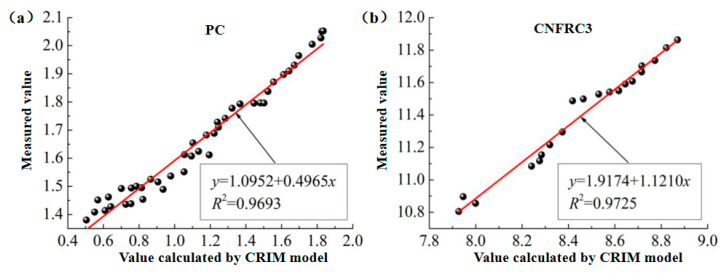
The fitting for relation between measured values and calculated values for ε^,,^ based on CRIM model. (**a**) PC; (**b**) CNFRC3.

**Figure 14:**
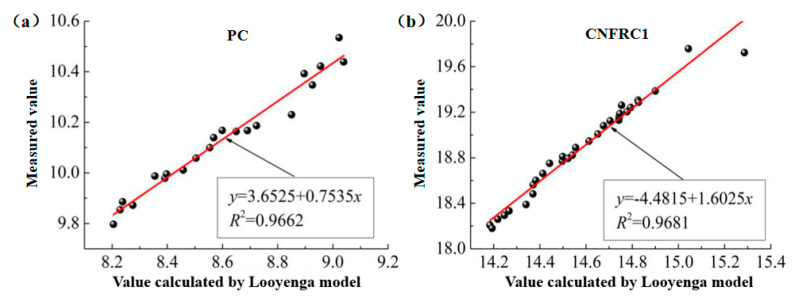
The fitting for relation between measured values and calculated values for ε^,^ based on Looyenga model. (**a**) PC; (**b**) CNFRC1.

**Figure 15:**
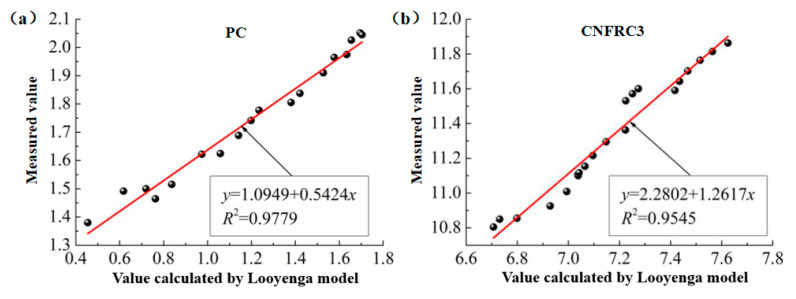
The fitting for relation between measured values and calculated values for ε^,,^ based on Looyenga model. (**a**) PC; (**b**) CNFRC1.

(1) The fitting for the relation between measured values and calculated values based on the Brown model was as shown in Figure 10 and Figure 11, respectively (only some groups were given).

(2) The fitting for the relation between measured values and calculated values based on the CRIM model was as shown in Figure 12 and Figure 13, respectively (only some groups were given).

(3) The fitting for the relation between measured values and calculated values based on the Looyenga model was as shown in Figure 14 and Figure 15, respectively (only some groups were given).

In Figure 10, Figure 11, Figure 12, Figure 13, Figure 14 and Figure 15, the y axis represents the measured value in the test, and the x axis represents the calculated value based on the models. Therefore, according to the above fitting, the modified formulas for calculation based on models can be obtained as follows.

The modified formula for calculation based on the Brown model is as follows.

(8) $$\frac{\varepsilon_{m}-b_{B}}{a_{B}}=v_{1}\varepsilon_{1}+v_{2}\varepsilon_{2}+v_{3}\varepsilon_{3}$$

The modified formula for calculation based on the CRIM model is as follows.

(9) $$\sqrt{\frac{\varepsilon_{m}-b_{C}}{a_{C}}}=v_{1}\sqrt{\varepsilon_{1}}+v_{2}\sqrt{\varepsilon_{2}}+v_{3}\sqrt{\varepsilon_{3}}$$

The modified formula for calculation based on the Looyenga model is as follows.

(10) $${\left(\frac{\varepsilon_{m}-b_{L}}{a_{L}}\right)}^{1/3}=v_{1}{\left(\varepsilon_{1}\right)}^{1/3}+v_{2}{\left(\varepsilon_{2}\right)}^{1/3}+v_{3}{\left(\varepsilon_{3}\right)}^{1/3}$$

According to the fitting results, the parameters for groups of specimens based on the modified Brown model, CRIM model and Looyenga model are summarized in Table 3 and Table 4.

### 3.4. Validation of Dielectric Model

**Figure 16:**
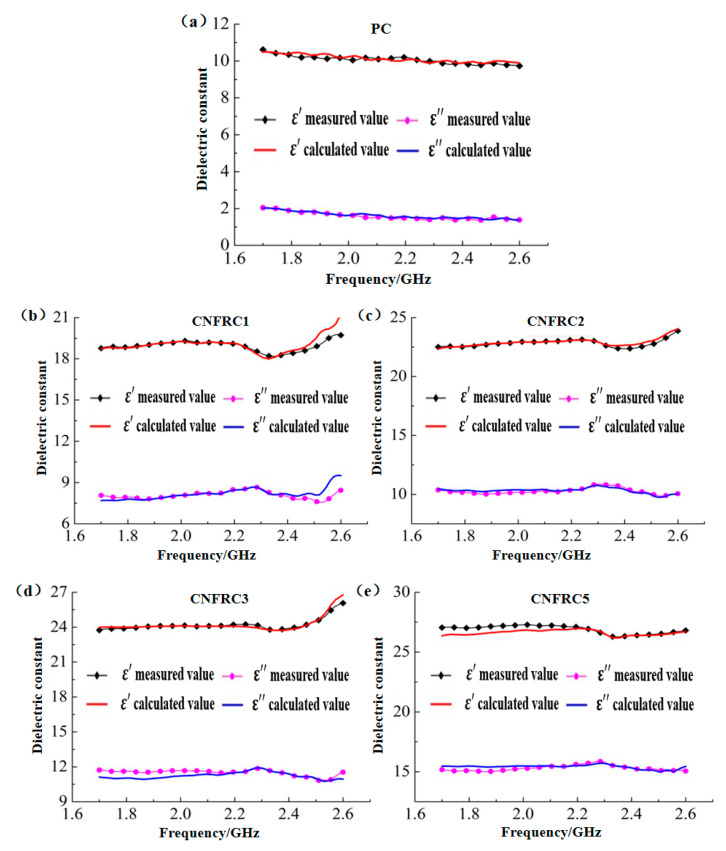
Verification of the measured values with the calculated values based on modified Brown model for groups of specimens. (**a**) PC; (**b**) CNFRC1; (**c**) CNFRC2; (**d**) CNFRC3; (**e**) CNFRC5.

The model curves for groups were obtained according to the modified Brown model in Formula (8) and verified with the measured curves, as shown in Figure 16.

**Figure 17:**
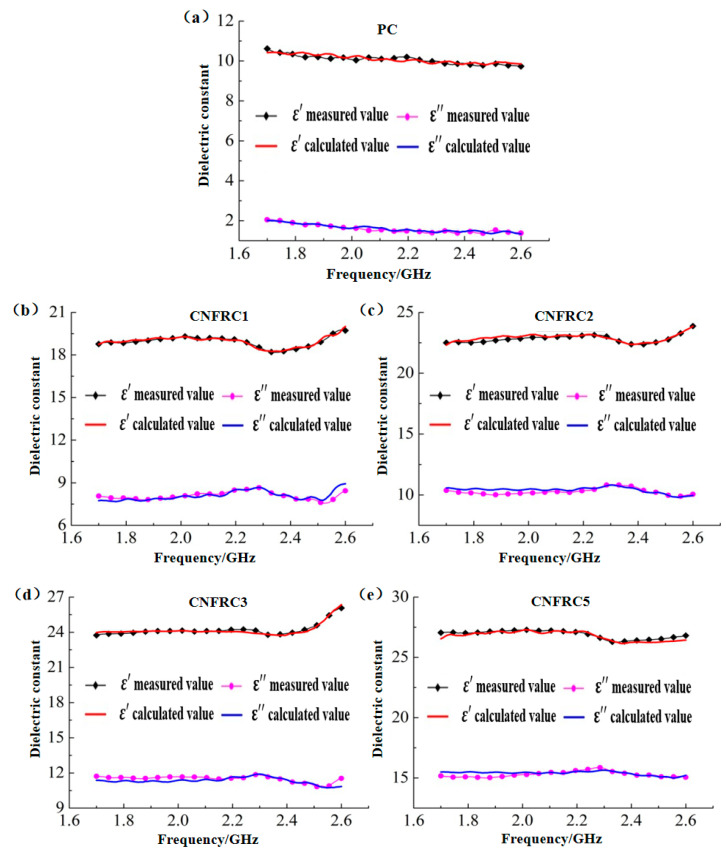
Verification of the measured values with the calculated values based on modified CRIM model for groups of specimens. (**a**) PC; (**b**) CNFRC1; (**c**) CNFRC2; (**d**) CNFRC3; (**e**) CNFRC5.

The model curves for groups were obtained according to the modified CRIM model in Formula (9) and verified with the measured curves, as shown in Figure 17.

**Figure 18:**
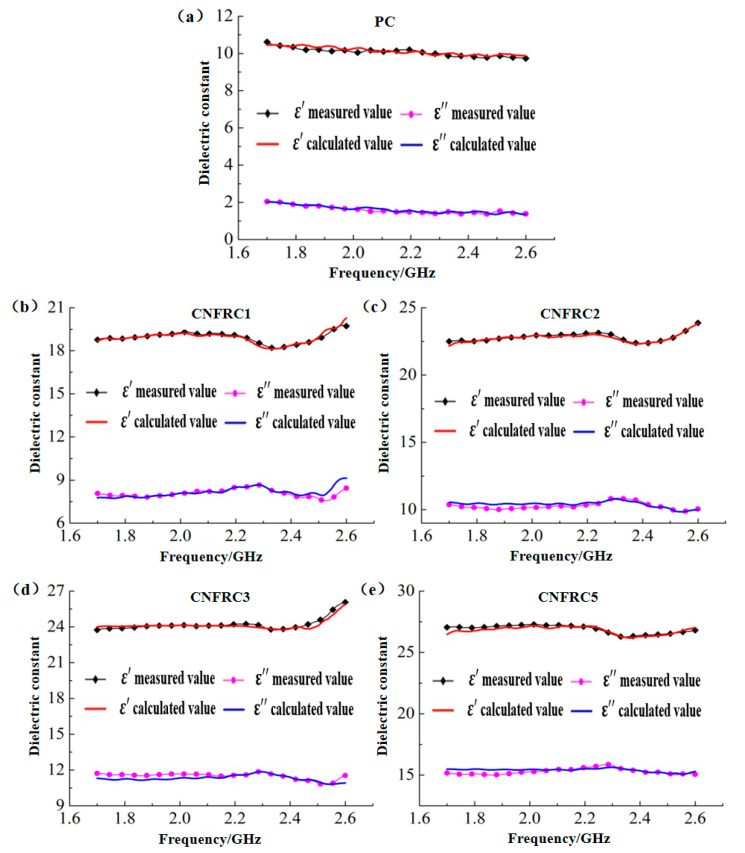
Verification of the measured values with the calculated values based on modified Looyenga model for groups of specimens. (**a**) PC; (**b**) CNFRC1; (**c**) CNFRC2; (**d**) CNFRC3; (**e**) CNFRC5.

The model curves for groups were obtained according to the modified Looyenga model in Formula (10) and verified with the measured curves, as shown in Figure 18.

According to Figure 16, Figure 17 and Figure 18, the goodness of fit between the measured values and the calculated values based on the modified Brown model, CRIM model and Looyenga model was high, and all of them accurately described the variation rule of the dielectric constant with the electromagnetic frequency for groups of specimens on the whole. The average errors for dielectric constants of models before and after modification was as shown in Table 5 and Table 6.

According to Table 5, the goodness of fit for *ε^,^* after modification of the models was greatly improved as compared with that before the modification. Only the average errors for *ε^,^* of the specimens in PC group and CNFRC5 group were more than 1.00%, but still below 1.20%, and the average errors for *ε^,^* of specimens in all the other groups were below 1.00%, with the minimum average error for *ε^,^* of 0.35%. On the whole, the *ε^,^* was improved by 1.88~3.75% with the modified Brown model. With the modified CRIM model, the improvement effect for *ε^,^* was the best in the CFNRC5 group. The *ε^,^* in the group was improved by 19.26%, and with modified Looyenga model, the *ε^,^* was improved by 13.60~25.83%. Therefore, the effect of the modified Looyenga model was the best.

According to Table 6, after the models were modified, the average errors for *ε^,,^* of the specimens in the PC group were still the largest, but within 3.55%. The improvement effect for errors of specimens was very good in all other groups, and the maximum average error for *ε^,,^* after modification was only 2.50% On the whole, with the modified Brown model, the *ε^,,^* was improved by 1.86~26.38%; with the modified CRIM model, the average errors for *ε^,,^* were controlled within 1.90% except for the PC group; with the modified Looyenga model, the improvement effect was still the best, and the *ε^,,^* was improved by 34.05~39.05%.

## 4. Conclusions

The transmission and reflection method was used to measure the dielectric constants of carbon nanofiber reinforced concrete as the fiber content was 0, 0.1%, 0.2%, 0.3% and 0.5%. Then, the modified dielectric constant models for the Brown model, CRIM model and Looyenga model were established based on the experimental data. Lastly, the modified models were verified for reliability, and a comparative analysis of errors before and after the modification was conducted. The conclusions are as follows.

(1) The carbon nanofiber reinforced concrete can be considered as composed of three independent phases, matrix (mortar), coarse aggregate (limestone gravel) and air, and the dielectric models of carbon nanofiber reinforced concrete with different degrees of fiber content can be effectively established based on the Brown model, CRIM model and Looyenga model.
(2) The goodness of fit between the calculated curves based on the three modified models and the measured curves was very high, and the variation rule for the dielectric constant of carbon nanofiber concrete with the frequency of electromagnetic wave can be described accurately.
(3) For the ε^,^ and ε^,,^ as dielectric constants calculated based on the modified models, the errors relative to corresponding measured values were effectively controlled. For the dielectric constant ε^,^, the average error was maintained below 1.2%, and the minimum error was only 0.35%. For the dielectric constant ε^,,^, the average error was maintained below 3.55%.

The results show that the three modified models can accurately describe the dielectric properties of carbon nanofiber reinforced concrete. Based on these models, qualitative or quantitative analysis and determination of moisture content, density, porosity and other aspects of carbon nanofiber reinforced concrete can be carried out based on the measured dielectric constant, providing data and technical support for the quality evaluation of existing concrete structures. Therefore, the determination of the dielectric model of carbon nanofiber reinforced concrete has important theoretical and application value, and it is recommended to study in depth and promote.

## Figures and Tables

**Table 1:** Mix proportion of carbon nanofiber reinforced concrete (kg/m^3^).

Specimen Number	Water Cement Ratio	Carbon Nanofiber	Cement	Water	Gravel	Sand	Water Reducing Agent	Defoamer
PC	0.36	0	495	180	1008	672	0	0
CNFRC1	0.36	0.30	495	180	1008	672	5.0	0.30
CNFRC2	0.36	0.45	495	180	1008	672	7.5	0.45
CNFRC3	0.36	0.60	495	180	1008	672	10.0	0.60
CNFRC5	0.36	0.90	495	180	1008	672	15.0	0.90

**Table 2:** Total pore volume of each specimen.

Specimen Number	PC	CNFRC1	CNFRC2	CNFRC3	CNFRC5
Total pore volume/mL·g^−1^	0.0445	0.0403	0.0384	0.0372	0.0395

**Table 3:** Parameter ε^,^ based on modified models.

Specimen No.	Modified Brown Model			Modified CRIM Model			Modified Looyenga Model		
	*A_b_*	*B_b_*	*R* ^2^	*A_b_*	*B_b_*	*R* ^2^	*A_b_*	*B_b_*	*R* ^2^
PC	3.8958	0.6512	0.9528	3.7408	0.7164	0.9148	3.6525	0.7535	0.9662
CNFRC1	10.9010	1.6415	0.9391	6.7750	1.6451	0.9917	4.4815	1.6025	0.9681
CNFRC2	1.8577	0.9560	0.9282	3.3751	1.4170	0.9908	4.8883	1.6272	0.9879
CNFRC3	13.9716	0.4290	0.8539	15.9737	0.4135	0.9663	15.8701	0.4523	0.9179
CNFRC5	7.8723	1.3218	0.8962	12.5804	1.8318	0.9582	10.8879	1.9125	0.9708

**Table 4:** Parameter ε^,,^ based on modified models.

Specimen No.	Modified Brown Model			Modified CRIM Model			Modified Looyenga Model		
	*a_B_*	*b_B_*	*R* ^2^	*a_B_*	*b_B_*	*R* ^2^	*a_B_*	*b_B_*	*R* ^2^
PC	1.0879	0.4701	0.9902	1.0952	0.4965	0.9693	1.0949	0.5424	0.9779
CNFRC1	0.1481	1.0650	0.9677	0.2006	1.4027	0.9826	1.1217	1.7709	0.9377
CNFRC2	0.3029	1.0773	0.9261	2.1980	1.0797	0.9692	2.8046	1.1607	0.9646
CNFRC3	1.3857	1.1472	0.9550	1.9174	1.1210	0.9725	2.2802	1.2617	0.9545
CNFRC5	5.1358	0.7032	0.9426	9.1860	0.5736	0.9246	9.5073	0.6436	0.8973

**Table 5:** Average error for ε^,^ of models before and after modification.

Specimen No.	Brown Model			CRIM Model			Looyenga Model		
	Before Modification/%	After Modification/%	Improvement Effect/%	Before Modification/%	After Modification/%	Improvement Effect/%	Before Modification/%	After Modification/%	Improvement Effect/%
PC	4.95	1.20	3.75	11.92	0.96	10.96	14.64	1.04	13.6
CNFRC1	3.68	0.91	2.77	17.51	0.43	17.08	22.78	0.38	22.4
CNFRC2	3.57	0.49	3.08	19.17	0.35	18.82	25.13	0.49	24.64
CNFRC3	3.66	0.46	3.2	19.79	0.53	19.26	25.97	0.39	25.58
CNFRC5	2.93	1.05	1.88	19.67	0.56	19.11	26.36	0.53	25.83

**Table 6:** Average errors for ε^,,^ before and after improvement of models.

Specimen No.	Brown Model			CRIM Model			Looyenga Model		
	Before Modification/%	After Modification/%	Improvement Effect/%	Before Modification/%	After Modification/%	Improvement Effect/%	Before Modification/%	After Modification/%	Improvement Effect/%
PC	29.85	3.47	26.38	36.15	3.34	32.81	40.89	3.55	37.34
CNFRC1	7.54	2.50	5.04	26.31	1.79	24.52	35.66	1.61	34.05
CNFRC2	3.96	1.29	2.67	26.04	1.69	24.35	36.22	1.84	34.38
CNFRC3	4.30	2.44	1.86	27.13	1.87	25.26	37.56	1.75	35.81
CNFRC5	4.45	1.15	3.3	29.09	1.12	27.97	40.16	1.11	39.05
